# Polymer-Doped SnO_2_ as an Electron Transport Layer for Highly Efficient and Stable Perovskite Solar Cells

**DOI:** 10.3390/polym16020199

**Published:** 2024-01-09

**Authors:** Vo Pham Hoang Huy, Chung-Wung Bark

**Affiliations:** Department of Electrical Engineering, Gachon University, Seongnam 13120, Gyeonggi, Republic of Korea; vophamhoanghuy@yahoo.com.vn

**Keywords:** perovskite solar cells, tin oxide, electron transport layers, polyacrylic acid, doping materials

## Abstract

To produce highly efficient and repeatable perovskite solar cells (PSCs), comprehending interfacial loss and developing approaches to ameliorate interfacial features is essential. Nonradiative recombination at the SnO_2_–perovskite interface in SnO_2_-based perovskite solar cells (PSCs) leads to significant potential loss and variability in device performance. To improve the quality of the SnO_2_ electron transport layer, a novel polymer-doped SnO_2_ matrix, specifically using polyacrylic acid, was developed. This matrix is formed by spin-coating a SnO_2_ colloidal solution that includes polymers. The polymer aids in dispersing nanoparticles within the substrate and is evenly distributed in the SnO_2_ solution. As a result of the polymer addition, the density and wetting properties of the SnO_2_ layer substantially improved. Subsequently, perovskite-based photovoltaic devices comprising SnO_2_ and Spiro-OMeTAD layers and using (FAPbI_3_)_0.97_(MAPbBr_3_)_0.03_ perovskite are constructed. These optimized devices exhibited an increased efficiency of 17.2% when compared to the 15.7% power conversion efficiency of the control device. The incorporation of polymers in the electron transport layer potentially enables even better performance in planar perovskite solar cells.

## 1. Introduction

Metal halide perovskite has emerged as a key technology for high-performance optoelectronic devices, owing to its long diffusion length and high defect tolerance [1,2,3,4]. Recent advancements have seen perovskite solar cells (PSCs) achieve a rapid increase in power conversion efficiency (PCE), reaching up to 26% [5,6,7]. This progress parallels the optimal standard of commercial silicon cells [8]. Planar PSCs are becoming increasingly prominent and competitive in the photovoltaic field due to their simple synthesis process, low cost, and high efficiency [9,10,11,12]. However, challenges such as significant photocurrent hysteresis and unstable output under operational conditions remain major concerns for many planar PSCs [13,14,15]. These issues are largely attributed to ion migration and suboptimal interfacial properties in the devices [16,17]. Many studies have underscored the importance of smooth and dense surfaces for efficient electron transport and effective hole blocking, which prevents holes from moving from the absorber layers to the transparent electrode [18,19]. Consequently, there is a strong consensus on the necessity of designing optimal electron transport.

The ETL plays a vital role in PSC performance. To inhibit carrier recombination, the ETL must simultaneously prevent the migration of carriers to the counter electrode and facilitate the charge transport process from the photoactive layer to the electrode. Specifically, TiO_2_, ZnO, Nb_2_O_5_, Zn_2_SO_4_, Fe_2_O_3_, In_2_O_3_, and SnO_2_ are widely used ETLs in PSCs. The specific performance of varied types of ETL is presented in detail in Table 1.

Despite an increase in the number of semiconductor materials that are being discovered and applied for ETL in PSC, TiO_2_ and SnO_2_ have been extensively used as ETLs due to their superior properties. Mesoporous TiO_2_ coated over compact TiO_2_ was initially used as the electron transport material for PSCs, originating from solid-state dye-sensitized solar cells [27]. Nevertheless, the low electron mobility of TiO_2_ as well as high-temperature annealing have both negative and detrimental effects on the future application of PSCs [28,29,30,31]. Additionally, the strong effects of photocatalytic TiO_2_ require special attention because they reduce the stability of PSC under illumination [32,33]. Recently, SnO_2_ has been widely used as the most promising ETL, with a proven efficiency of over 21% and a substantial reduction in photocatalytic and hysteresis issues under illumination [34,35]. SnO_2_ exhibits the typical properties of an ideal ETL with high mobility [up to 240 cm^2^/(V·s)], large bandgap (exceeding 3.6 eV), and excellent chemical stability, making it a strong candidate for highly efficient PSCs [36,37]. However, SnO_2_ is inherently an insulating material when processed at low temperatures, and the efficiency of the device is heavily dependent on the thickness of the SnO_2_ layer [38,39,40,41]. To achieve fast charge transfer, high-performance SnO_2_-based PSCs require a thin SnO_2_ layer that is less than 30 nm in thickness. The aggregation issue of the nanoparticles in the film makes it difficult to fabricate such a thin and compact layer using the spin-coating technique [42,43,44]. Typically, the resulting SnO_2_ thin film has extensive areas of nonuniformity and pinholes. Additionally, pretreatment (such as UV treatment) is necessary to change the poor wetting performance of SnO_2_ with the perovskite precursor solution.

Doping organic compounds into electron transport layers (ETLs) can either align the Fermi level of the ETL with the conduction band of perovskite or influence the perovskite/hole transport layers, improving the crystallization and grain size of the perovskite layer [45,46,47]. Thus, doping effectively lowers trap defects in the photo absorber and ETL, enhancing charge separation and transfer for efficient perovskite solar cell performance. Based on this approach, polyethylenimine ethoxylate (PEIE) was utilized to enhance the electron transport capacity of SnO_2_ by suppressing trap-assisted recombination and lowering the energy barrier between the active layer, as well as improving the wetting ability of these layers, resulting in high PSC performance [48]. Additionally, incorporating polyethylene glycol (PEG) promoted the separation of nanoparticles in the film and significantly improved the density and wetting properties of the SnO_2_ layer, leading to an improvement in device performance [49]. Furthermore, polyvinyl pyrrolidone (PVP), another water-soluble polymer, was also used for this purpose [50]. The formation of bonds between the electron pairs of nitrogen (or oxygen) in PVP and metal ions enables PVP to adhere to metal surfaces, while the long vinyl polymer chain of the PVP backbone prevents nanoparticle agglomeration through steric hindrance [50].

Given these challenges, doping the ETL with polymers to create a uniform and compact SnO_2_ ETL and enhance the PCE of devices is valuable. PAA polymer has been extensively used in various energy storage technologies, such as electrolytes in flexible symmetrical supercapacitors, novel separators in silver oxide batteries, or as binders for GaP anodes in lithium-ion batteries [51,52,53]. However, the use of PAA as a doping material in SnO_2_ colloidal precursors for PSCs has not been explored. We propose that integrating PAA with the SnO_2_ colloidal solution could help in forming an effective ETL. Additionally, we present the development of an ideal PAA@SnO_2_ ETL, combining SnO_2_ with Li-ion. By selecting water-soluble polymers such as PAA, which dissolve in the SnO_2_ colloidal aqueous solution, we discovered that the PAA chain effectively limits the aggregation of SnO_2_ nanoparticles in the precursor solution. This combination with polymers substantially improves the quality of the film, enhancing its compaction and wetting properties.

## 2. Experiment Section

### 2.1. Materials

Lead Iodide (PbI_2_), Lithium chloride (LiCl), spiro-oMeTAD (99.8%), methylammonium bromide (MABr), methylammonium chloride (MACl), chlorobenzene (CB), isopropanol (IPA), DMSO, DMF, acetonitrile (ACN), lithium bis(trifluoromethanesulfonyl) imide (Li-TFSI), and 4-tert-butyl were purchased from Sigma-Aldrich (Seoul, Republic of Korea). A SnO_2_ colloidal solution (15 wt% in water) was purchased from Alfa Aesar (Seoul, Republic of Korea). Formamidinium iodide (FAI) was purchased from GreatCell Solar (Queanbeyan, Australia).

### 2.2. Perovskite Solar Cell Fabrication

**Figure 1:**
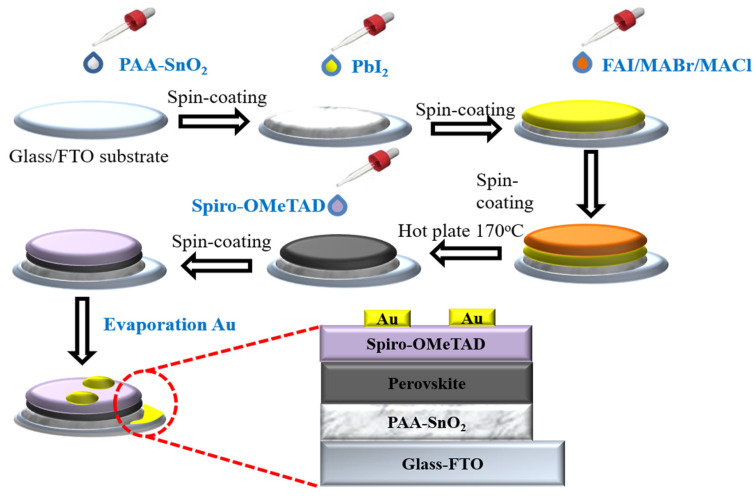
Schematic illustrating the process of preparing complete solar cell.

The preparation of PSC is shown in detail in Figure 1. The glass/FTO substrate was cleaned using distilled water, acetone, and isopropanol. Then, the FTO glasses were dried using a nitrogen gun and treated with UV–ozone for 20 min. The SnO_2_ precursor was obtained by dissolving SnO_2_ (450 μL) colloid dispersion and LiCl (300 μL) aqueous solution (17 mg/4 mL) in water (2 mL) [49]. For PAA@SnO_2_, PAA (0.5, 1.0, 2.0, and 3.0 mg/mL) was added into the SnO_2_ precursor solution. The SnO_2_-based electron transport layer was spin-coated on the cleaned FTO at 3000 rpm for 30 s. This was followed by annealing at 150 °C for 30 min in air. Then, the substrates were treated with UV–ozone for 20 min before being transferred into a glovebox. The perovskite films were fabricated using a typical two-step sequential deposition method. First, 50 μL of PbI_2_ precursor solution (600 mg PbI_2_ dissolved in (DMF 0.9 mL) and (DMSO 0.1 mL)) was spin-coated onto SnO_2_ films at 2000 rpm for 20 s. The FAI/MABr/MACl mixed solution (60 mg FAI, 6 mg MABr, and 6 mg MACl dissolved in 1 mL IPA) was spin-coated onto the PbI_2_ film at 4000 rpm for 20 s. Then, the films were annealed at 150 °C for 20 min. After cooling to room temperature, the hole-transport layer was subsequently deposited on top of the perovskite film via spin-coating at 4000 rpm for 40 s using a chlorobenzene solution, which contained Spiro-OMeTAD (72.3 mg mL^−1^), tert-butylpyridine (29 μL mL^−1^), and bis(trifluoromethane)sulfonimide lithium salt (18.5 μL mL^−1^, 520 mg mL^−1^ in acetonitrile). Finally, 100 nm of gold electrodes were deposited on top of the devices via evaporation at approximately 10^−6^ Torr.

### 2.3. Characterization

To assess the ability of film to absorb light, the UV–Vis spectrophotometer Agilent (Varian, Cary 50, San Diego, CA, USA) was used to measure the UV–Vis light-absorption spectra of film. The X-ray diffraction (XRD) patterns were acquired using an XRD Rigaku DMAX 2200 system (Tokyo, Japan) with Cu K as the X-ray source (λ = 0.15406 nm). The Fourier transform infrared (FTIR) spectra were captured using an infrared spectrometric analyzer (Vertex 70, Bruker, Karlsruhe, Germany). Field emission scanning electron microscopy (FESEM, Hitachi S-4700, Tokyo, Japan), operating at 10 kV, was used to analyze the top and cross-sectional morphologies of the samples. Using a QuantaMaster TM 50 PTI (Piscataway, NJ, USA), steady-state photoluminescence (PL) spectra were acquired. A solar simulator (McScience K3000, Solar Simulator LAB 50, and Polaromix, Gyeonggi, Republic of Korea) simulating solar radiation with an irradiance of 100 mW cm^−2^ (AM 1.5 G) was used to model sunlight conditions. The external quantum efficiency (EQE) was determined using a McScience K3100 measurement system (Suwon, Republic of Korea).

## 3. Results and Discussion

Despite semiconductor oxide nanoparticles being recognized as potential electron transport layer (ETL) materials for perovskite solar cells (PSCs), their tendency to agglomerate due to van der Waals forces within the colloid, coupled with their large specific surface area, presents a significant challenge [54,55]. Various strategies, including doping and surface engineering, have been implemented to curb the agglomeration of SnO_2_ particles in dispersion systems with small-sized particle media, thereby stabilizing SnO_2_ colloids. The suspension, uniformity, and stability of nanoparticles in water are heavily influenced by particle spatial agglomeration and charge balance. Introducing a suitable polymer or surfactant material helps to reduce inter-particle space and maintain a uniform charge on the particle surfaces, thus preventing oxide nanoparticle agglomeration. In this context, PAA is used as a buffering agent to stabilize SnO_2_ particles, working through two mechanisms: (i) PAA reduces the available active space around the particles, preventing SnO_2_ nanoparticle agglomeration, and (ii) crucially, functional groups on the PAA surface, when combined with SnO_2_ nanoparticles, help balance the charge between particles. To explore the impact of PAA on the photovoltaic characteristics and enhance PSC performance, a range of material characterization techniques were applied, as detailed throughout this manuscript.

**Figure 2:**
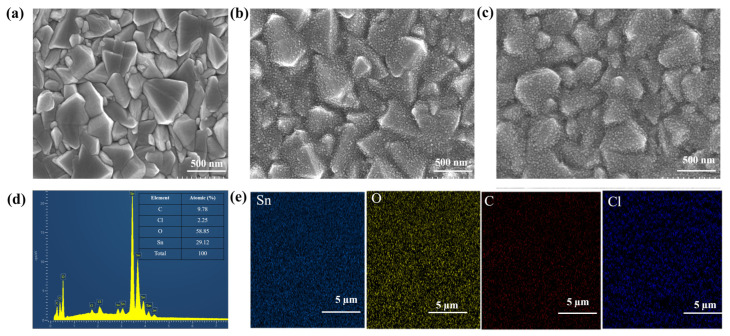
SEM image of (**a**) pristine FTO, (**b**) FTO/SnO_2_, (**c**) FTO/PAA@SnO_2_, (**d**) EDX spectrum of PAA@SnO_2_, and (**e**) elemental mapping corresponding to PAA@SnO_2_ on FTO/glass.

We initially investigated the surface morphology obtained on different ETLs (SnO_2_ and PAA@SnO_2_) (as shown in Figure 2). Compared with the FTO surface (Figure 2a), SnO_2_ and PAA@SnO_2_ were successfully prepared (Figure 2b,c). However, the PAA@SnO_2_ nanocrystals were densely coated, and there were no pinholes on the film surface. This can prevent ineffective contact between the ETL film surface and perovskite, leading to improved optical performance. The EDX analysis presents the existing components on the PAA@SnO_2_ film (Figure 2d). The presence of a small amount of residual Cl (2.25%) does not significantly affect the composition of the film due to the guaranteed elemental ratio between Sn and O (specifically SnO_2_) along with the existence of PAA on the FTO substrate. Furthermore, Figure 2e shows the elemental mapping spectra corresponding to PAA@SnO_2_ film, showing the uniform distribution of Sn, O, and C nanocrystals throughout the film.

**Figure 3:**
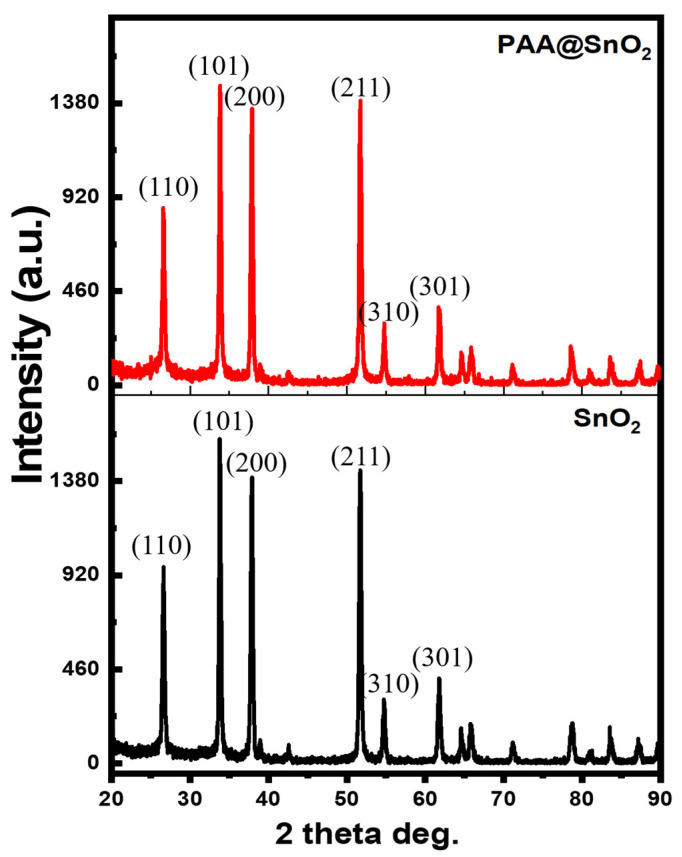
XRD pattern of SnO_2_ and PAA@SnO_2_.

XRD analysis was performed to obtain crystallographic information regarding as-synthesised pure SnO_2_ and PAA@SnO_2_. Figure 3 displays the acquired XRD patterns for PAA@SnO_2_ and pristine SnO_2_. The main diffraction peaks at approximately 26.25°, 33.66°, 38.16°, 51.50°, 54.82°, and 61.55° for pure SnO_2_ correspond to (110), (101), (200), (211), (310), and (301), respectively. This indicates that a pure rutile tetragonal SnO_2_ structure has formed (JCPDS no. 41–1445). When a low concentration of PAA was added as a dopant, no additional peak was observed in the PAA@SnO_2_, indicating that PAA doping did not introduce a secondary phase as an impurity with the pure SnO_2_. Conversely, a reduction in peak intensity was observed in PAA@SnO_2_ spectrum, likely resulting from the interaction between the PAA polymer and SnO_2_ crystals.

**Figure 4:**
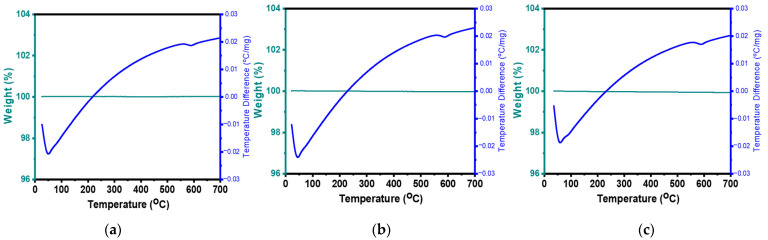
Thermal properties of (**a**) FTO, (**b**) FTO/SnO_2_, and (**c**) FTO/PAA@SnO_2_.

Thermogravimetric analysis (TGA) and differential scanning calorimetry (DSC) were used to determine the thermal properties of FTO, FTO/SnO_2_, and FTO/PAA@SnO_2_ from 25 °C to 700 °C in nitrogen gas flowing at a rate of 10°/min. Figure 4a shows that the FTO substrate remains stable in N_2_ up to 700 °C. This indicates good thermal stability without pyrolysis or thermo-oxidative decay occurring. However, conducting investigations at temperatures above 700 °C is not advisable, as this can lead to a decrease in Hall mobility and reduction in the number of oxygen vacancies. These changes result in lower mobility and fewer carriers in the FTO membrane, ultimately leading to the increased resistivity of the FTO substrate [56]. Additionally, the spin-coating of SnO_2_ and PAA@SnO_2_ forms thin films of 69 and 35 nm, respectively. Thus, the mass loss is not too significant, specifically 99.98% (Figure 4b) and 99.94% (Figure 4c). Furthermore, the obtained curve of the DSC analysis is very similar. In Figure 4a–c, the DSC plot shows two endothermic peaks. The first endothermic peak is approximately in the range of 45–110 °C, corresponding to the removal of the absorbed water from the substrate [57,58]. The other small peak at 580 °C was due to further crystallinity of the FTO substrate [59,60]. Therefore, the degree of SnO_2_ and PAA@SnO_2_ crystallization on the FTO substrate is appropriate for the annealing temperature of 150 °C.

**Figure 5:**
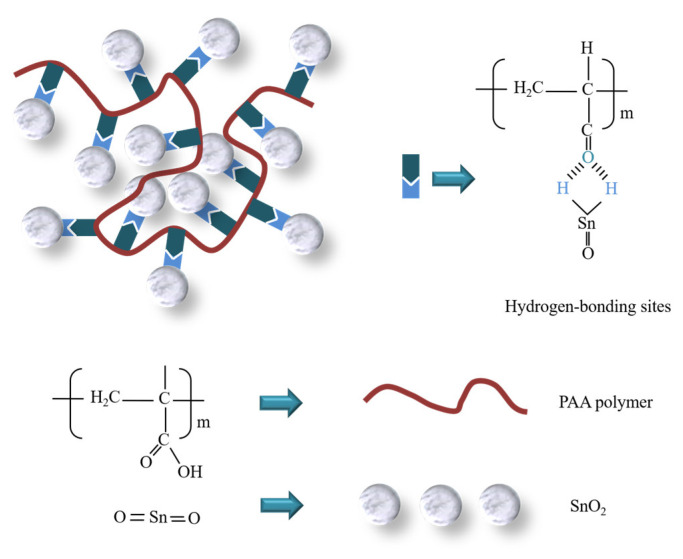
Chemical structure of SnO_2_ and PAA@SnO_2_.

Next, to investigate the influence as well as find the optimal concentration of PAA, we adjusted the PAA concentration to 0.5, 1.0, 2.0, and 3.0 mg mL^−1^, respectively. The undoped or doped SnO_2_ ETL were spin-coated with SnO_2_ precursor solution at ambient temperature and then thermally annealed in air for 30 min at 170 °C. The chemical structure of PAA-SnO_2_ is shown in Figure 5.

**Figure 6:**
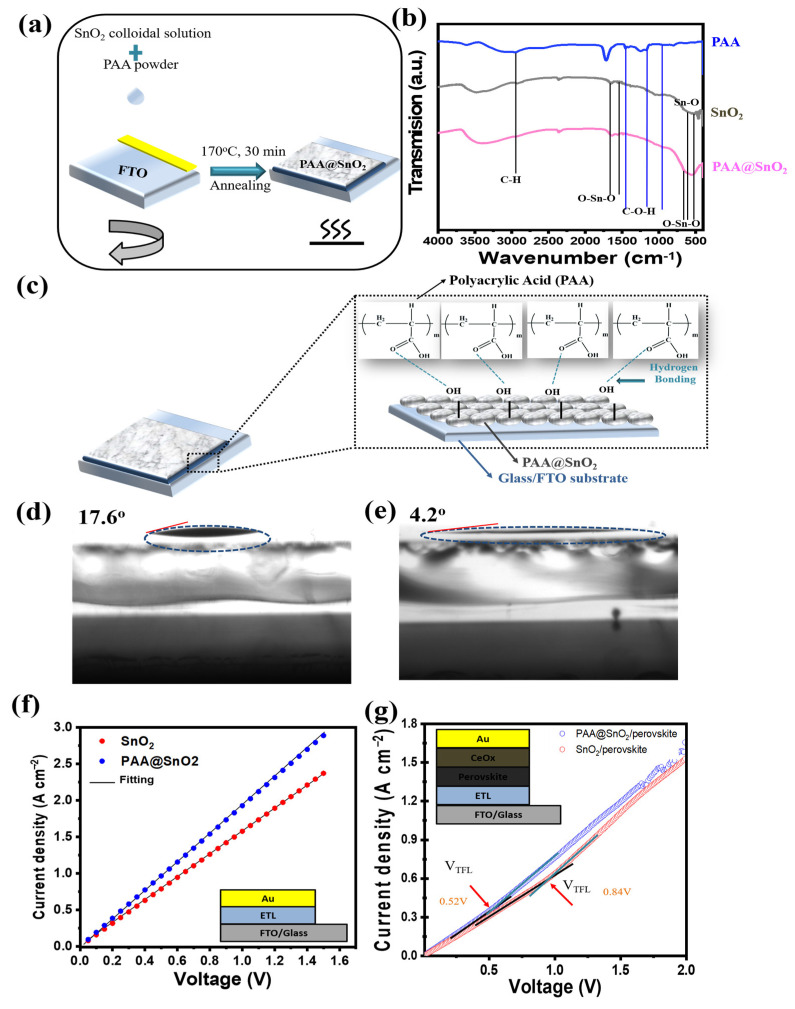
(**a**) Schematic illustrating the pristine and PAA@SnO_2_ ETL preparation. (**b**) FT-IR spectra of PAA, SnO_2_, and PAA@SnO_2_ films before annealing. (**c**) Diagram of mechanism for hydrogen bond formation. Contact angle measurement of SnO_2_ films without (**d**) or with (**e**) PAA polymer (blue circle: boundary region to determine the contact angle). (**f**) The electron mobility for the SnO_2_ and PAA@SnO_2_ films calculated by the SCLC model with the device structure of Glass/FTO/ETL/Au. (**g**) Dark I-V measurement of the electron-only devices based on SnO_2_ and PAA@SnO_2_ ETLs.

Figure 6a shows a facile synthesis of ETL used to collect and transport electrons from the absorber layer to the TCO. First, the ability to transmit in the visible region of pristine SnO_2_ and PAA@SnO_2_ at different concentrations was evaluated. From Figure S1a, undoped and doped SnO_2_ ETL show excellent transmittance in the wavelength region from 300–900 nm, and the transmittance of the PAA@SnO_2_ at various film concentrations almost remains unchanged when compared with that of the pristine SnO_2_ film. This indicates that the addition of PAA slightly changes the optical transmittance properties of the undoped SnO_2_ film. Additionally, a good transmittance spectrum is advantageous to the performance of PSCs because it shows uniform film deposition with few defect states. However, a minor decrease in the transmittance of the ETL is observed, corresponding to the optical image of the solution shifting from transparent to opalescent with the increasing doping concentration of PAA, as illustrated in Figure S1b. Additionally, the absorbance progressively increases as the wavelength increases, as shown in Figure S2a. The Tauc plot (Figure S2b) was utilized to estimate the optical band edge (E_g_). It was determined that the E_g_ of the pristine SnO_2_ film was 4.2 eV. This value remained nearly unchanged for PAA@SnO_2_, suggesting that the small amount of PAA doping did not significantly impact the E_g_ of the pristine SnO_2_ film.

Fourier transform infrared (FT-IR) spectroscopy was analyzed to determine the presence of separate components in the PAA@SnO_2_ film before annealing (Figure 6b). The O-Sn-O stretching mode and Sn-O vibrational mode are responsible for the absorption peaks at 550, 650, 1550, and 1690 cm^−1^, respectively [49]. The COOH stretching modes in PAA are responsible for the peaks to appear at 1479, 1296, and 948 cm^−1^. It can be seen that PAA was absorbed into SnO_2_ film by noting that the spectrum of PAA@SnO_2_ exhibits the typical peaks of SnO_2_ and PAA, with the O-Sn-O peaks of SnO_2_ shifting to 670 cm^−1^. Furthermore, the presence of PAA at different concentrations in the SnO_2_ precursor solution was demonstrated by the FT-IR spectrum (Figure S3). More importantly, we explored whether annealing plays a role in the formation of PAA@SnO_2_. According to Figure S4, there is a slight change in the composition of pristine SnO_2_ and PAA@SnO_2_ at various concentrations post-annealing. Notably, the presence of hydroxyl groups on the film surface at 2850 and 2915 cm^−1^ indicates strong hydrogen bonding with the carboxylic group in the PAA polymer. This is attributed to the high temperature of the annealing process, which promotes the partial breakdown of the Sn=O bond, leading to the formation of Sn(OH)n on the SnO_2_ surface. The mechanism of this hydrogen bond formation is depicted in Figure 6c. Therefore, during the annealing process, the hydroxyl group on the SnO_2_ surface forms a hydrogen bond with the carboxylic group of PAA (as shown in Figure 5) [49,53,61]. 

By suppressing the formation of large clusters in the substrate and using SnO_2_ that has been incorporated into PAA via hydrogen bonding, it is anticipated that the spin-coated film will become compact and uniform with a decrease in pinholes and that the PAA polymer will create a dense and compact matrix on the substrate. Additionally, to obtain complete coverage on the substrate, UV pretreatment is required because pure SnO_2_ film is a non-wettable surface for a perovskite precursor solution. This implies that the water contact angle measurement of pristine SnO_2_ is high (approximately 40°) [62,63] and clearly decreases after UV treatment (17.6°). Fortunately, PAA inclusion in SnO_2_ film can enhance the affinity between SnO_2_ and perovskite by altering the SnO_2_ surface, as demonstrated in Figure 6d,e and Figure S5. Therefore, increasing PAA concentration will contribute to further reducing contact angle of SnO_2_. However, excessively increasing the PAA concentration (specifically 3 mg/mL) will promote particle aggregation, leading to a rebound increase in contact angle data. When compared to PAA@SnO_2_ at various concentrations, the contact angle of the perovskite solution on pure SnO_2_ film was significantly higher. Additionally, perovskite is not completely coated on the virgin SnO_2_ substrate without pretreatment. However, perovskite on PAA@SnO_2_ displays a dense and compact coating (Figure S6). Hence, without any pretreatment (such as UV treatment), a complete covering of perovskite film on the PAA@SnO_2_ layer is accomplished.

The effect of PAA on the conductivity of the SnO_2_ thin films was analyzed via electrical measurements. As shown in Figure 6f, the PAA@SnO_2_ film exhibits a higher electron mobility (2.08 × 10^−3^ cm^2^ V^−1^ s^−1^) than that of the SnO_2_ film (8.5 × 10^−4^ cm^2^ V^−1^ s^−1^) as measured by the space charge limited current (SCLC) method [36,64]. The SCLC model was used with the electron-only devices (FTO/ETL/perovskite/CeO_x_/Au) to investigate the trap density [65]. The dark I-V curves for the two devices are displayed in Figure 6g. Typically, the I-V curve exhibits a linear ohmic-type response at low bias voltages. The current begins to increase nonlinearly with an increase in bias voltage, signaling the commencement of the trap-filling process. Furthermore, trap-filled limit voltage (V_TFL_) is the definition of the kink point that separates the linear and nonlinear regions. The trap density (*N_t_*) can be computed using the following equation:

$$N_{t}=\frac{2\;{ɛ}_{0}ɛ\;V_{TFL}}{e\;L^{2}}$$

where ɛ_0_, ɛ, *e*, and *L* denote the perovskite film thickness, elementary charge, relative dielectric constant, and permittivity of vacuum, respectively. The perovskite layer on the PAA@SnO_2_ ETL has an estimated trap density of approximately 6.27 × 10^14^ cm^−3^, which is significantly less than the 4.4 × 10^15^ cm^−3^ of the film deposited on the SnO_2_ ETL, indicating that the introduction of PAA reduces the defect states of the ETL.

**Figure 7:**
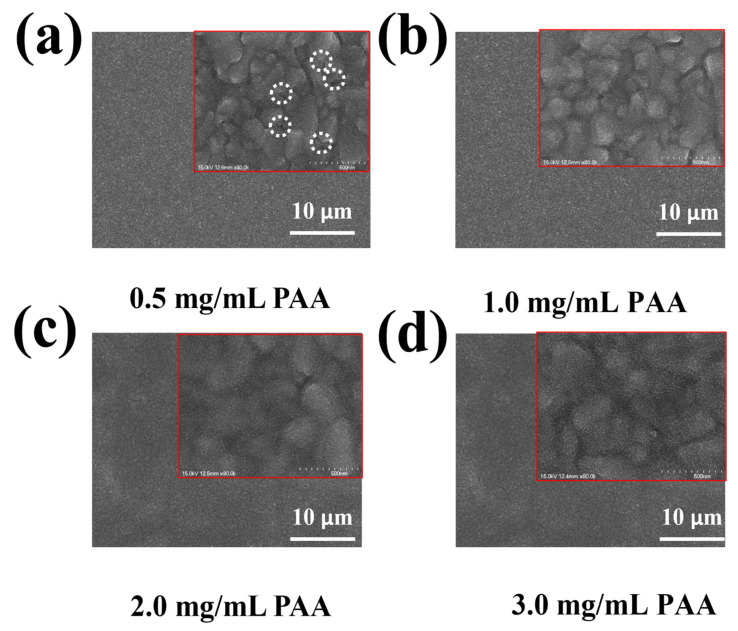
Top-view SEM image of (**a**) pristine SnO_2_ (dashed circles: pinholes on the surface) and (**b**–**d**) PAA@SnO_2_ at various concentrations (red squares: high-magnification SEM image).

With or without PAA doping, compact SnO_2_ films were spin-coated onto FTO substrates using a colloid dispersion solution. To better understand the surface morphology of the coated films, atomic force microscopy (AFM) was employed. Given that FTO shows a roughness surface of 30 nm [66,67], both SnO_2_ ETL films exhibit a smooth surface, as observed in Figure S7a,b. However, a pristine SnO_2_ film exhibits a rougher surface than the SnO_2_ ETL films (26.32 nm vs. 24.98 nm), which is in line with the surface morphology observed via scanning electron microscopy (SEM), as shown in Figure 2b,c. Furthermore, it should be observed that at a concentration of 0.5 mg mL^−1^, several pinholes are visible, despite the fact that the agglomeration between particles is significantly reduced when compared to pristine SnO_2_ (as shown in Figure 7a). This result suggests that the SnO_2_ oligomers can be disaggregated by the hydrogen bonds of PAA and SnO_2_ particles. However, their affinity is insufficient to glue the particles together. When the concentration increases slightly (1.0 mg mL^−1^), the PAA@SnO_2_ surface is pinhole-free with no intergranular agglomeration through uniform particle formation (Figure 7b). Additionally, due to the high hydrogen bonding affinity between PAA and SnO_2_, which occurred when the mass concentration of PAA exceeded 1.0 mg mL^−1^ or when there were more PAA components present, PAA@SnO_2_ aggregated into large particles and failed to form a uniform and dense ETL layer in Figure 7c–d.

**Figure 8:**
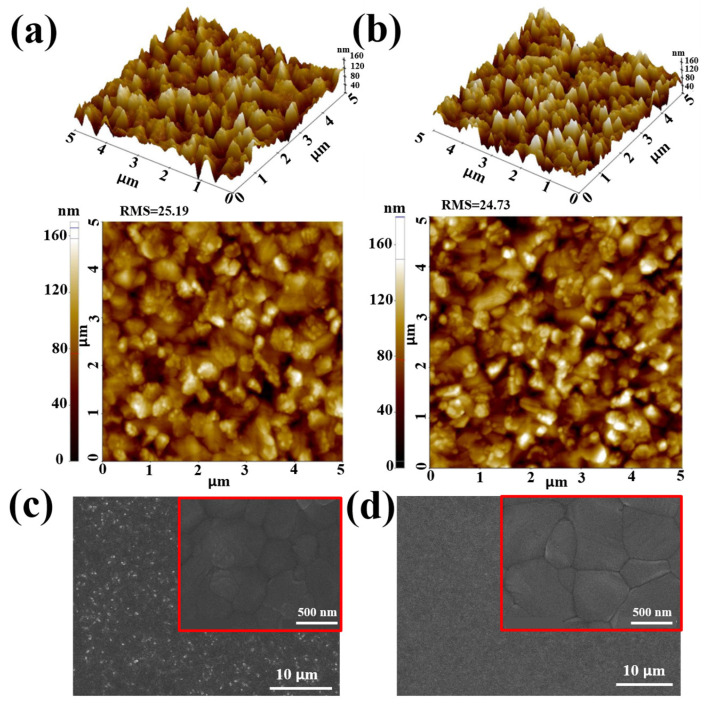
AFM images of perovskite coated on (**a**) SnO_2_ and (**b**) PAA@SnO_2_ layer. Top-view of SEM images of perovskite coated on (**c**) SnO_2_ and (**d**) PAA@SnO_2_ films.

AFM measurements revealed that the root mean square (RMS) roughnesses of perovskite films on SnO_2_ and PAA@SnO_2_ were 25.19 nm and 24.73 nm, respectively. This smoother perovskite is shown in Figure 8a,b. The SEM images of the absorber layer deposited onto various ETLs are shown in Figure 8c,d. The images confirm the surface morphology of the perovskite layers. The perovskite coated on PAA@SnO_2_ was also discovered to be bigger than that coated on the SnO_2_ layers. Additionally, as shown in Figure S8, the effects of various PAA concentrations in a SnO_2_ colloidal solution on the surface morphology of perovskite layers were also obtained. It can be determined that the crystal grains of the perovskite on PAA@SnO_2_ (0.5 and 1.0 mg/mL) are more uniform with less white PbI_2_ phase in comparison with those of the perovskite on pristine SnO_2_ and PAA@SnO_2_ (2.0 and 3.0 mg/mL). The result indicates that PAA@SnO_2_ can facilitate a uniform distribution of nucleation sites. Given that the incorporation of PAA leads to reduced roughness in the ETL thin film, the presence of a pre-existing functional group (carboxylic acid) on the PAA surface readily bonds with SnO_2_ to form hydrogen bonding, leading to nanoparticle disaggregation as well as the enhanced wetting properties of ETL for perovskite precursor solution.

By adjusting the PAA concentration and layer thickness, the performance of the device is maximized. To successfully enable electron transport for ETL, the concentration of insulating PAA in SnO_2_ should be maintained to a minimum. According to Table S1, the PCE of devices increases as PAA concentration increases, and the fill factor achieves its maximum when PAA content reaches 1.0 mg mL^−1^. Conversely, there was a decrease in device PCE as the concentration of PAA increased (e.g., 2.0 and 3.0 mg mL^−1^), which is thought to decrease the presence of SnO_2_ nanoparticles in solution. Furthermore, the high affinity between PAA and SnO_2_ contributes significantly to the formation of nanoparticle aggregations. Additionally, due to the weak conductivity of SnO_2_ produced at low temperatures, the layer thickness should be as thin as possible while still preventing significant current leakage. For both pure SnO_2_ and PAA@SnO_2_, a well-defined boundary between each layer indicates thick coatings on the substrate, as shown in Figure S9a,b. Notably, the PAA@SnO_2_ layer is significantly thinner (approximately 35 nm) when compared to pure SnO_2_ (approximately 69 nm). The polymer matrix reduces the thickness of the ETL by approximately 50%, a substantial reduction when compared to the majority of ETLs reported in literature [68,69], which can enhance ETL light transmission and decrease series resistance. Furthermore, the thicknesses of the perovskite, spiro-OMeTAD, and metal electrode (Au) layers are approximately 560 nm, 195 nm, and 30 nm, respectively. Additionally, cross-section SEM images show that the surface roughness of pristine SnO_2_ is higher than that of PAA@SnO2 (Figure S9c,d). This highlights the advantages of using polymer doping materials, especially those with functional groups on their surfaces, such as PAA in this study.

**Figure 9:**
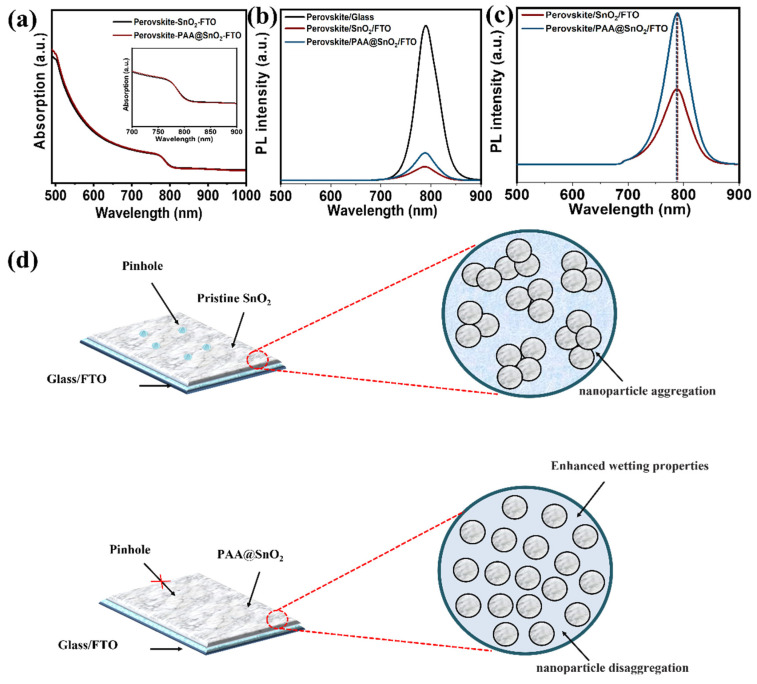
(**a**) UV−Vis absorption data of absorber films with SnO_2_ and PAA@SnO_2_ layers (the inset is amplifying absorption spectra in the wavelength range of 700−900 nm). (**b**,**c**) Steady-state PL spectra. (**d**) The diagram of film morphology for SnO_2_ and PAA@SnO_2_.

UV–Vis spectroscopy was used to assess the light-harvesting capabilities of perovskites with varied SnO_2_ and PAA@SnO_2_ ETLs (Figure 9a). Evidently, the PAA@SnO_2_ layer has a minor impact on the perovskite layer’s capacity for absorption. Nevertheless, its bandgap has not changed. From the Tauc plot in Figure S10, the value of Eg is estimated to be 2.05 eV, and there is not much difference in Eg between PAA@SnO_2_ and pristine SnO_2_. Therefore, doping PAA does not significantly affect the pristine SnO_2_ structure for the formation of the perovskite layer. Figure 9b,c show the steady-state photoluminescence (PL) of the absorber produced on different films. The PL peaks of the perovskite on SnO_2_ and PAA@SnO_2_ can be shown to have been significantly quenched, demonstrating a perfect band-edge alignment of SnO_2_ with perovskite (Figure 9b). The PL intensity of the perovskite film on the surface of the PAA@SnO_2_ film is greater than that observed on the SnO_2_ film under the identical test conditions. The perovskite layer on PAA@SnO_2_ may have undergone less nonradiative recombination due to the stronger PL intensity [70,71,72,73]. Additionally, the perovskite film’s PL peak on the PAA@SnO_2_ ETL shifts by around 1.1 nm to a lower wavelength, which signals fewer defects (Figure 9c) [74]. Using PAA-incorporated SnO_2_, the performance of PSC will be increased for two reasons, according to an examination of the characteristics of PAA@SnO_2_ when compared to virgin SnO_2_ (Figure 9d). First, pinholes can be minimized for film thicknesses under 40 nm by inhibiting nanoparticle aggregation, resulting in a more compact and homogeneous surface. Second, the PAA polymer can enhance the substrate’s wetting properties.

**Figure 10:**
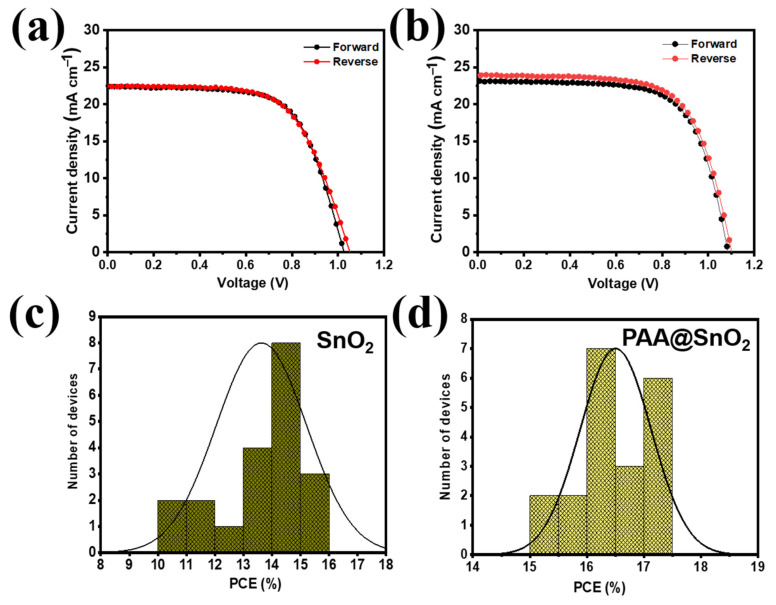
Current density−voltage (J−V) curves of devices based on (**a**) pristine SnO_2_ and (**b**) PAA@SnO_2_ with PAA concentration of 1.0 mg/mL under reverse-forward scanning directions. The PCE distribution of the PSCs of (**c**) SnO_2_ and (**d**) PAA@SnO_2_.

Based on the aforementioned advantages, the photovoltaic properties of undoped and doped-based SnO_2_ PSCs were evaluated. The J−V curves for SnO_2_ and PAA@SnO_2_-based devices in the reverse and forward scan directions are shown in Figure 10a,b and Figure S11. Table 2 illustrates the PAA@SnO_2_-based devices with high efficiency and J−V characteristics of the champion devices using ETLs. The devices based on the SnO_2_ ETL substrate exhibit a maximum PCE of 15.7%, with the specific values of Voc = 1.06 V, Jsc = 22.39 mA cm^−2^, and FF = 0.68. Surprisingly, by switching the control ETL to a PAA@SnO_2_ ETL, the detailed parameters of Voc and FF were considerably enhanced to 1.08 V and 0.73%, respectively. Furthermore, the optimal PCE can be increased to 17.2%. This study shows that interfacial carrier recombination was significantly reduced by the PAA@SnO_2_ layer’s ability to passivate charge recombination at the ETL/perovskite interfaces. For each undoped and PAA-doped ETL, we created 20 different devices to test the repeatability of the material and technique, as shown in Table S2. The distribution of device parameters is shown in Figure 10c,d together with the statistical parameters that are presented in Figure S12. The J–V values for the doped SnO_2_-based devices clearly show a narrow distribution with a low standard deviation, indicating exceptional repeatability.

**Figure 11:**
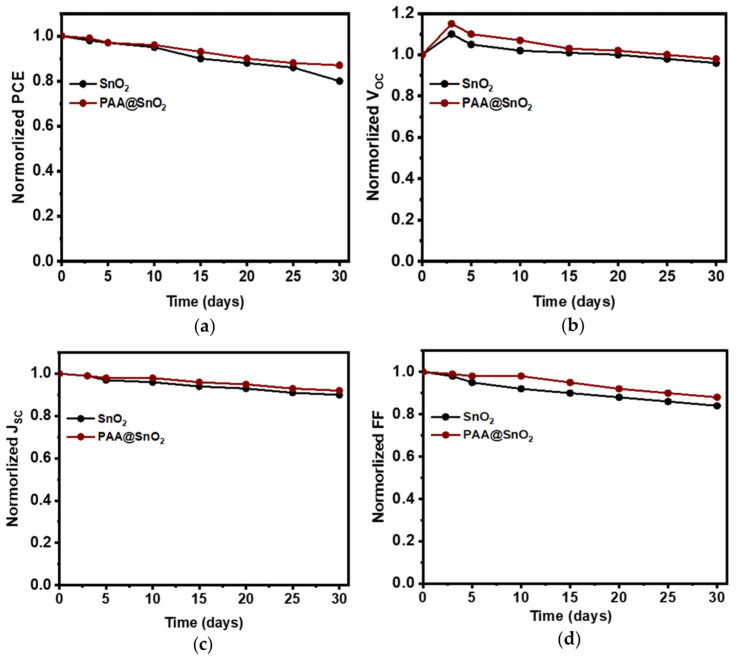
Shelf-stability of perovskite solar cells in a glove box without any encapsulation. Normalized (**a**) PCE, (**b**) V_OC_, (**c**) J_SC_, and (**d**) FF.

The long-term stability of PSCs with SnO_2_ and PAA@SnO_2_ was evaluated in a nitrogen environment without encapsulation. Figure 11 illustrates the shelf stability of PSCs with different ETLs over time. After 30 days of storage, the device using the PAA@SnO_2_ ETL retained 89% of its original power conversion efficiency (PCE), whereas the device with the SnO_2_ ETL experienced a 21% reduction in its initial PCE. This indicates that devices with the PAA@SnO_2_ ETL are more stable, likely due to improved contact between the PAA@SnO_2_ ETL and perovskite layer. The degradation of the charge carrier transport layer and perovskite layer is a well-known factor contributing to the instability of PSCs. The enhanced stability in the PAA@SnO_2_-based devices when compared to those with SnO_2_ can be attributed to the improved interaction between the ETL and the perovskite layer.

Table 3 summarizes the performance of PSCs with different doping materials in SnO_2_ ETL. When compared to the performance of other recent PSCs, our PAA@SnO_2_ exhibits performance increases from 15.7% to 17.2% (increase of approximately 10% in performance). It can be observed that doping with organic materials, specifically polymers, is slightly more effective than doping with inorganic materials due to the large particle size of the material, which easily forms large clusters on the ETL surface, contributing to reducing the surface contact between ETL and perovskite.

## 4. Conclusions

In summary, a straightforward and cost-effective technique has been suggested for the incorporation of PAA into the SnO_2_ matrix to create a high-quality electron-selective layer. The PAA@SnO_2_ layer, characterized by its dense and uniform structure with an ultra-thin thickness below 40 nm, is attributed to the enhanced dispersibility of the SnO_2_ substrate. Due to the enhanced wetting properties of the polymer-modified film, a pinhole-free and high-quality perovskite film is easily formed on the PAA@SnO_2_ matrix. Effective PSCs based on the PAA@SnO_2_ matrix have been demonstrated, achieving a power conversion efficiency of 17.2% with high repeatability and shelf stability. This study presents a novel perspective on the inclusion of polymers as electron/hole selective layers in colloidal quantum dot inks. It is anticipated that future research and development involving other inorganic nanoparticle inks combined with suitable polymers will enhance device performance. This study highlights a new approach in the simple combination of polymers with metal oxides, acting as an effective electron/hole selective layer. The development of different polymers with suitable metal oxides is expected to further improve the current performance of devices. This process is promising in its simplicity and effectiveness, promoting its potential widespread application on an industrial scale.

## Figures and Tables

**Table 1:** Device structure and performance parameters of different metal oxide ETLs.

ETL	Structure of Device	PCE (%)	V_OC_ (V)	J_SC_ (mA cm^−2^)	FF	Ref
TiO_2_	FTO/TiO_2_/MAPbI_3_/Spiro-OMeTAD/Au	20.9	1.17	23.31	0.77	[20]
ZnO	ITO/ZnO/PBDB-T:ITIC/MoO_3_/Ag	16.9	0.88	14.6	0.63	[21]
Nb_2_O_5_	ITO/NiO_x_/FA_0_._85_MA_0_._15_PbI_2_._55_Br_0_._45_/Nb_2_O_5_/Ag	18.3	1.08	22.7	0.72	[22]
Zn_2_SO_4_	ITO/Zn_2_SnO_4_/PCBM/CH_3_NH_3_PbI_3_/Spiro-OMeTAD/Ag	14.5	1.07	21.2	0.62	[23]
Fe_2_O_3_	FTO/Fe_2_O_3_/CH_3_NH_3_PbI_3_/Spiro OMeTAD/Au	10.78	0.65	16.6	0.63	[24]
In_2_O_3_	FTO/In_2_O_3_/CH_3_NH_3_PbI_3_/Spiro-OMeTAD/Au	13.97	1.07	19.3	0.68	[25]
SnO_2_	FTO/SnO_2_/(FAPbI_3_)_0_._95_(MAPbBr_3_)_0_._05_/Spiro-OMeTAD/Au	20.6	1.08	24.42	0.78	[26]

**Table 2:** Photovoltaic parameters of champion PSCs based on SnO_2_ and PAA@SnO_2_ ETLs.

Devices	Scan Direction	PCE (%)	J_sc_ (mA cm^−2^)	V_oc_ (V)	FF
SnO_2_-PSC	Reverse	15.7	22.39	1.06	0.68
	Forward	15.2	21.85	1.03	0.66
PAA@SnO_2_-PSC	Reverse	17.2	24.92	1.08	0.73
	Forward	16.8	23.12	1.07	0.69

**Table 3:** Comparison of the performance of PAA@SnO_2_ ETL in PSC with those of varying doping materials.

ETL	PCE (%)	V_OC_ (V)	J_SC_ (mA cm^−2^)	FF	Ref.
Pristine SnO_2_ Ta-doped SnO_2_	19.48 20.80	1.158 1.161	21.7 22.8	0.78 0.79	[75]
Pristine SnO_2_ Zn-doped SnO_2_	15.31 17.78	1.078 1.098	23.2 23.4	0.61 0.69	[76]
Pristine SnO_2_ Ga-doped SnO_2_	12.5 17.0	0.997 1.070	22.1 22.8	0.57 0.70	[77]
Pristine SnO_2_ Nb-doped SnO_2_	12.32 13.53	0.88 0.92	22.8 24.1	0.61 0.61	[78]
Pristine SnO_2_ La-doped SnO_2_	14.24 17.08	1.060 1.090	20.7 21.8	0.65 0.72	[79]
Pristine SnO_2_ Y-doped SnO_2_	11.69 15.60	1.030 1.070	18.6 21.8	0.61 0.67	[80]
Pristine SnO_2_ Cl-doped SnO_2_	15.07 18.10	1.020 1.110	21.0 23.0	0.59 0.69	[81]
Pristine SnO_2_ Al-doped SnO_2_	9.02 12.10	1.000 1.030	16.8 19.4	0.53 0.58	[65]
Pristine SnO_2_ KF-doped SnO_2_	13.40 15.39	1.180 1.310	14.6 14.8	0.78 0.79	[71]
Pristine SnO_2_ PVP-doped SnO_2_	18.05 19.42	1.100 1.130	21.0 21.1	0.79 0.81	[50]
Pristine SnO_2_ PEG-doped SnO_2_	18.60 20.80	1.070 1.110	22.6 22.7	0.77 0.82	[49]
Pristine SnO_2_ PAA-doped SnO_2_	15.70 17.20	1.06 1.08	22.4 24.9	0.68 0.73	This work

## Data Availability

Data are contained within the article and supplementary materials.

## Supplementary Materials

The following supporting information can be downloaded at: https://www.mdpi.com/article/10.3390/polym16020199/s1.
