# Supplementary material for: Polymer-Doped SnO2 as an Electron Transport Layer for Highly Efficient and Stable Perovskite Solar Cells
Source: Polymers (Basel). 2024 Jan 9;16(2):199. doi: 10.3390/polym16020199 (PMC10819156; doi:10.3390/polym16020199)
Supplement: Supplementary file 1 [file polymers-16-00199-s001.zip › polymers-2803385-supplementary.pdf]

SUPPORTING INFORMATION

Polymer-doped SnO_2 as an Electron Transport Layer for High-efficient and Stable Perovskite Solar Cells

Vo Pham Hoang Huy and Chung Wung Bark*

Department of Electrical Engineering, Gachon University, Seongnam 13120, Gyeonggi, Republic of Korea;

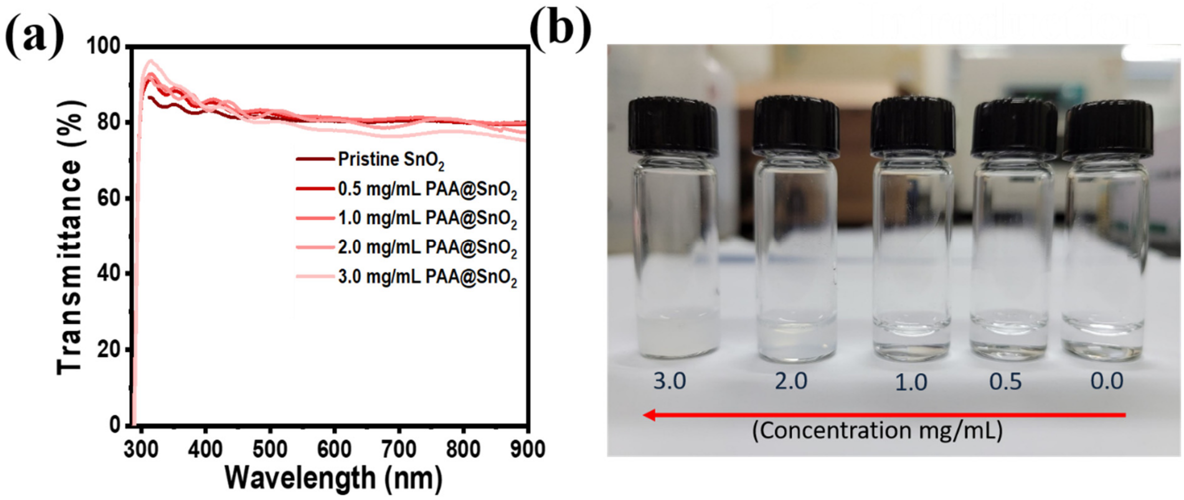

Fig. S1. (a) Transmission spectra of 0, 0.5, 1, 2, and 3.0 mg mL^{-1} of PAA@SnO_2 films coated onto FTO substrates. (b) Digital images of SnO_2 and different concentrations of PAA@SnO_2 precursor solutions.

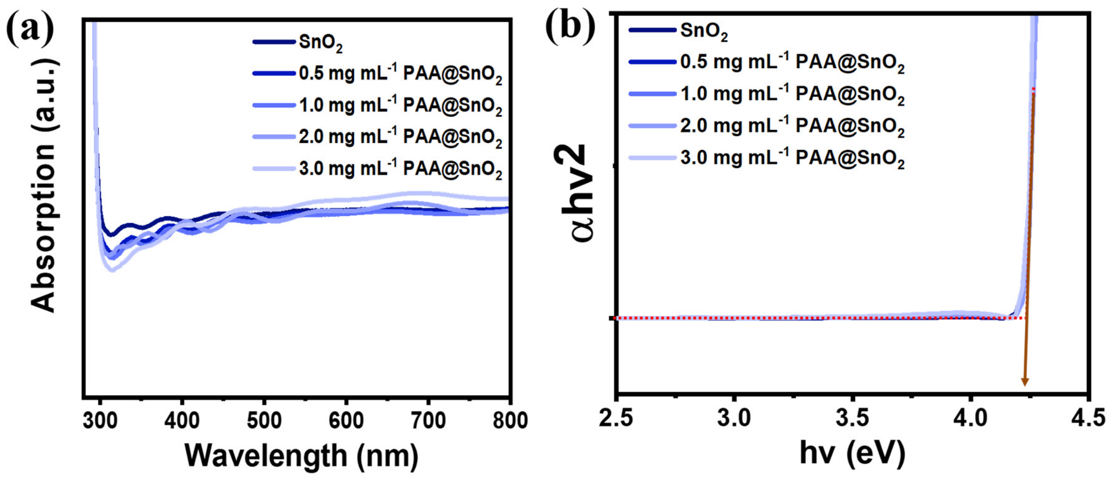

Fig. S2. (a) UV-Vis spectra of 0, 0.5, 1, 2, and 3.0 mg mL^{-1} of PAA@SnO_2 films coated on FTO substrates. (b) Tauc plot for PAA@SnO_2

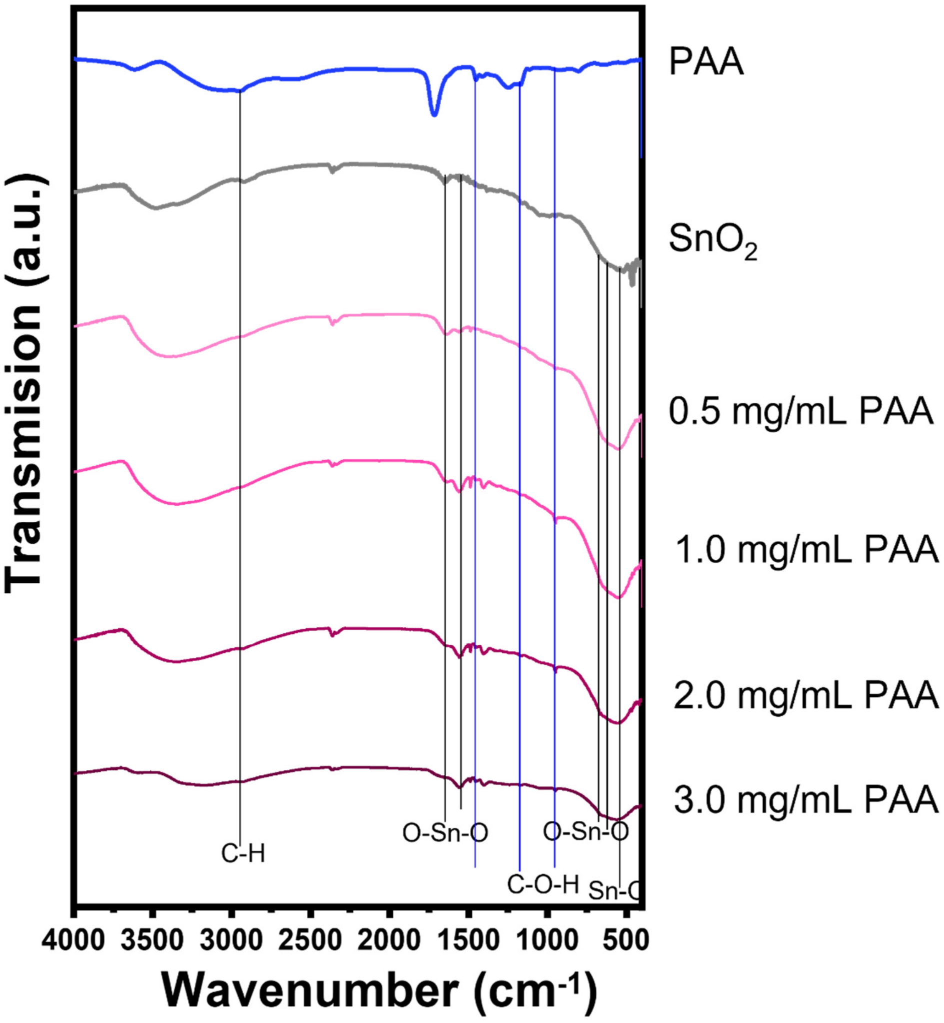

Fig. S3. FT-IR spectra of PAA, SnO_2, and PAA@SnO_2 at different concentrations before annealing.

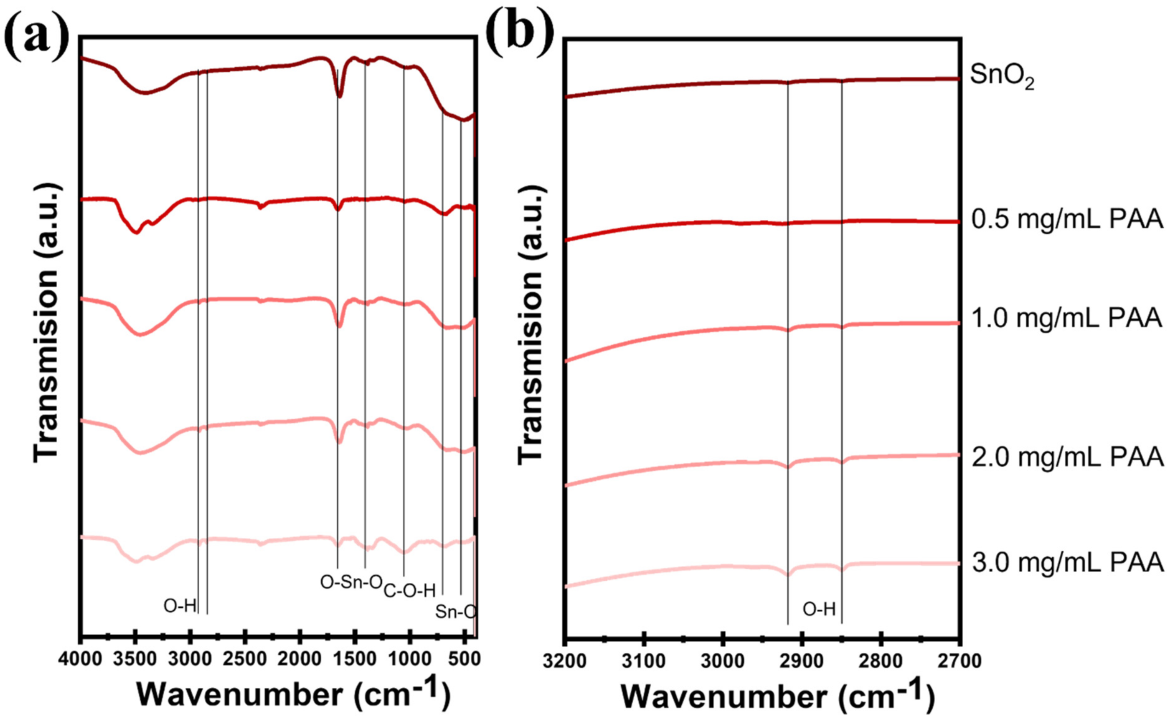

Fig. S4. FT-IR spectra of PAA, SnO_2, and PAA@SnO_2 at different concentrations after annealing.

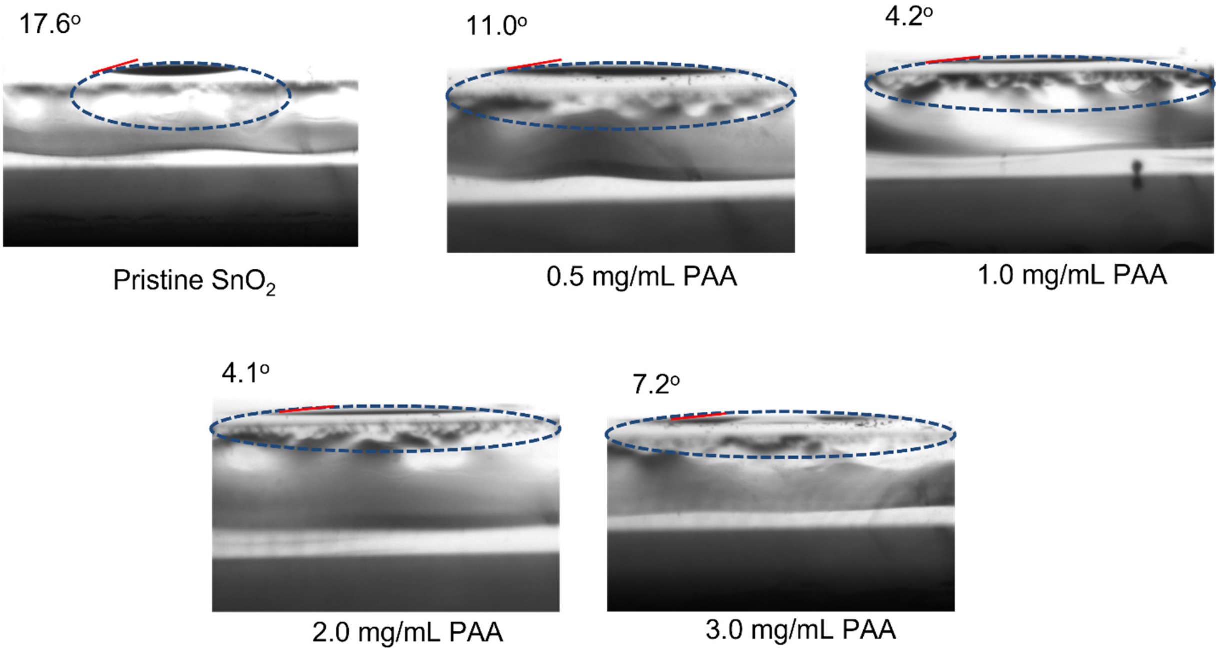

Fig. S5. Contact angle measurements of the SnO_2 films without and with different concentrations of the PAA polymer.

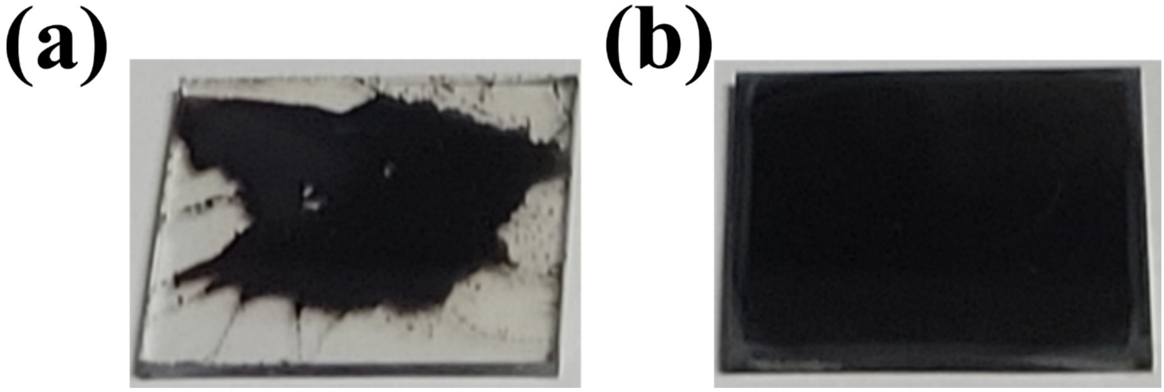

Fig. S6. Photograph of the perovskite film on (a) SnO_2 with the UV treatment and (b) PAA@SnO_2 with PAA concentration of 1 mg/mL substrate without the UV treatment.

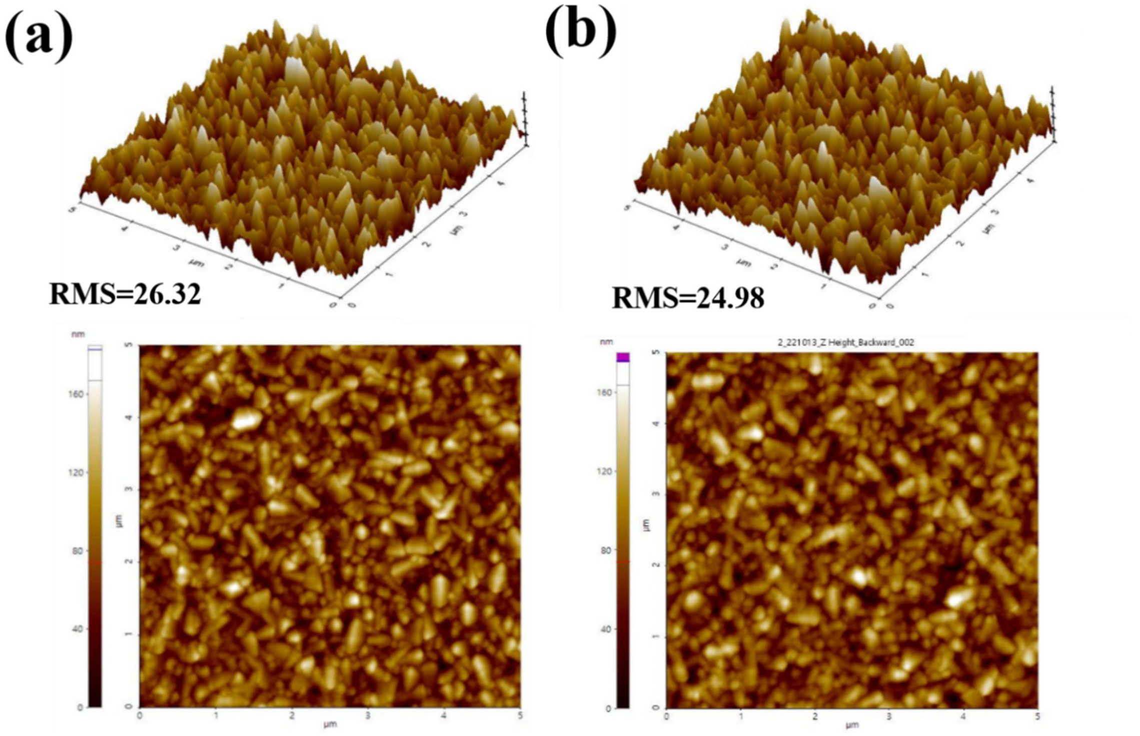

Fig. S7. AFM images of (a) SnO_2 and (b) PAA@SnO_2 layers.

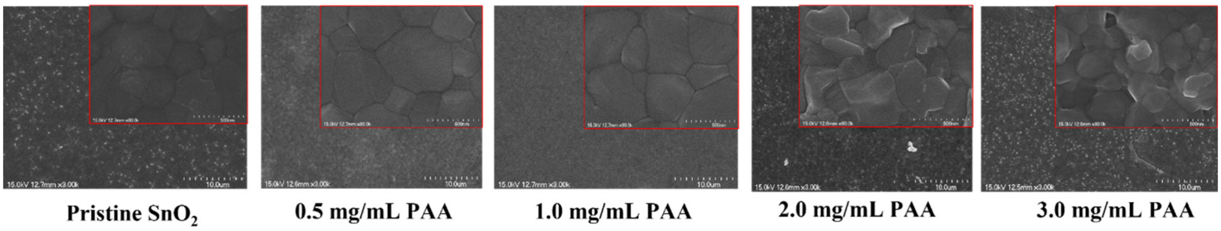

Fig. S8. SEM image of the perovskite films grown on pristine SnO_2 and PAA@SnO_2 at various concentrations.

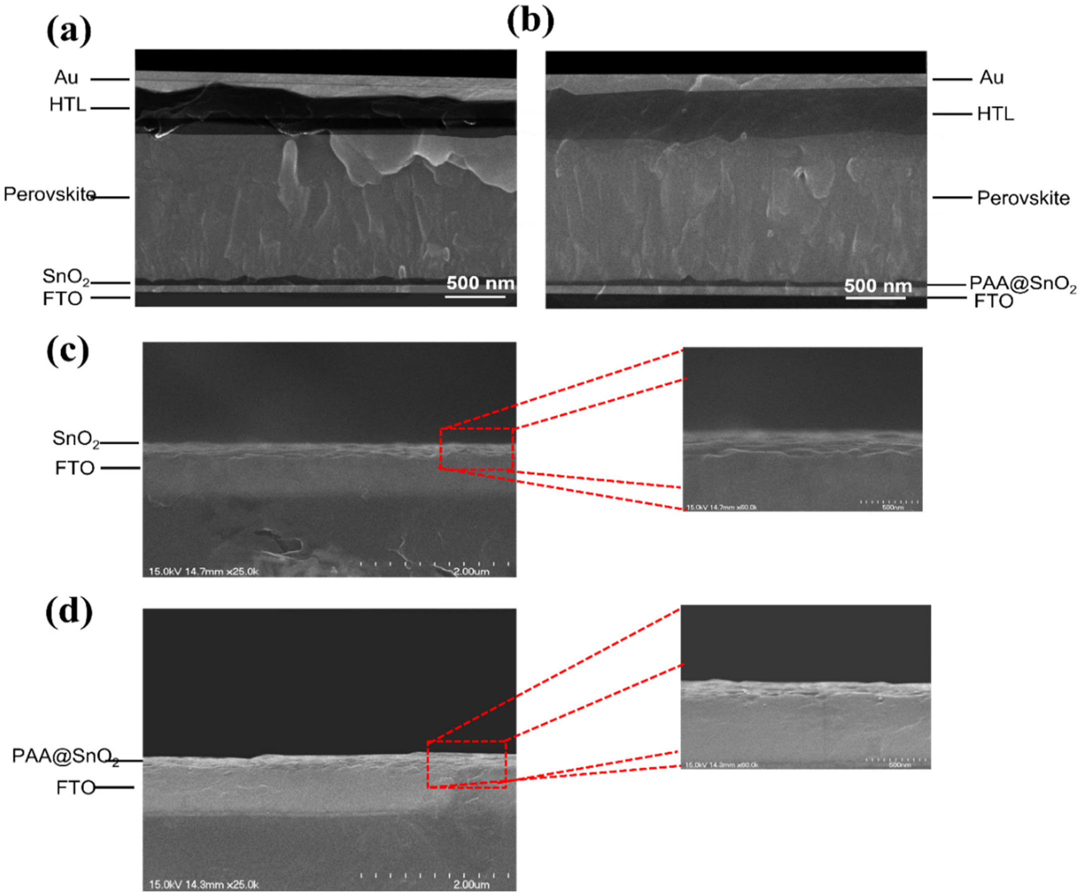

Fig. S9. Cross-section SEM images of (a) pristine SnO_2 and PAA@SnO_2 device structure. Cross-section SEM images of ETL with (a) pristine SnO_2 and PAA@SnO_2.

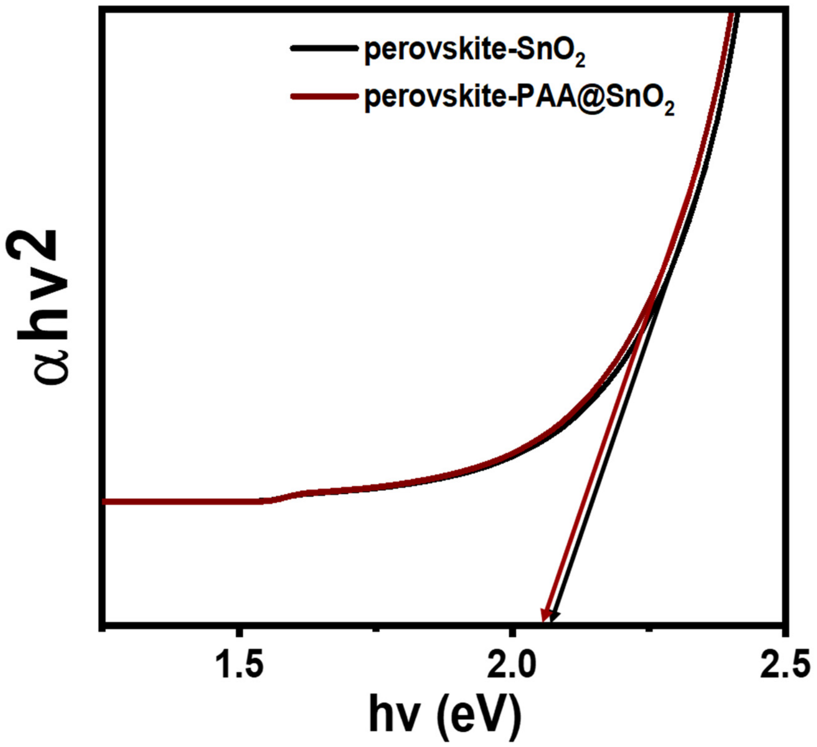

Fig. S10. Tauc plot for perovskite on SnO_2 and PAA@SnO_2

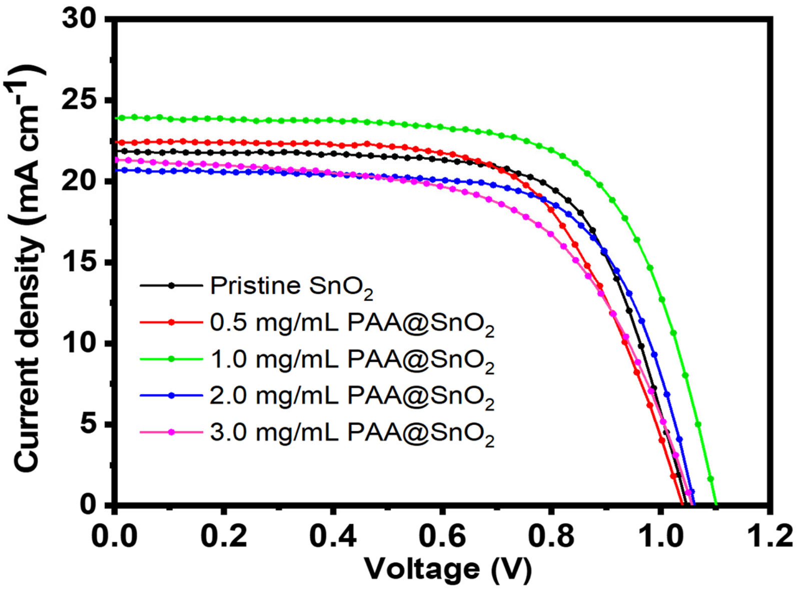

Fig. S11. J–V curves of the PSCs with different ETL substrates.

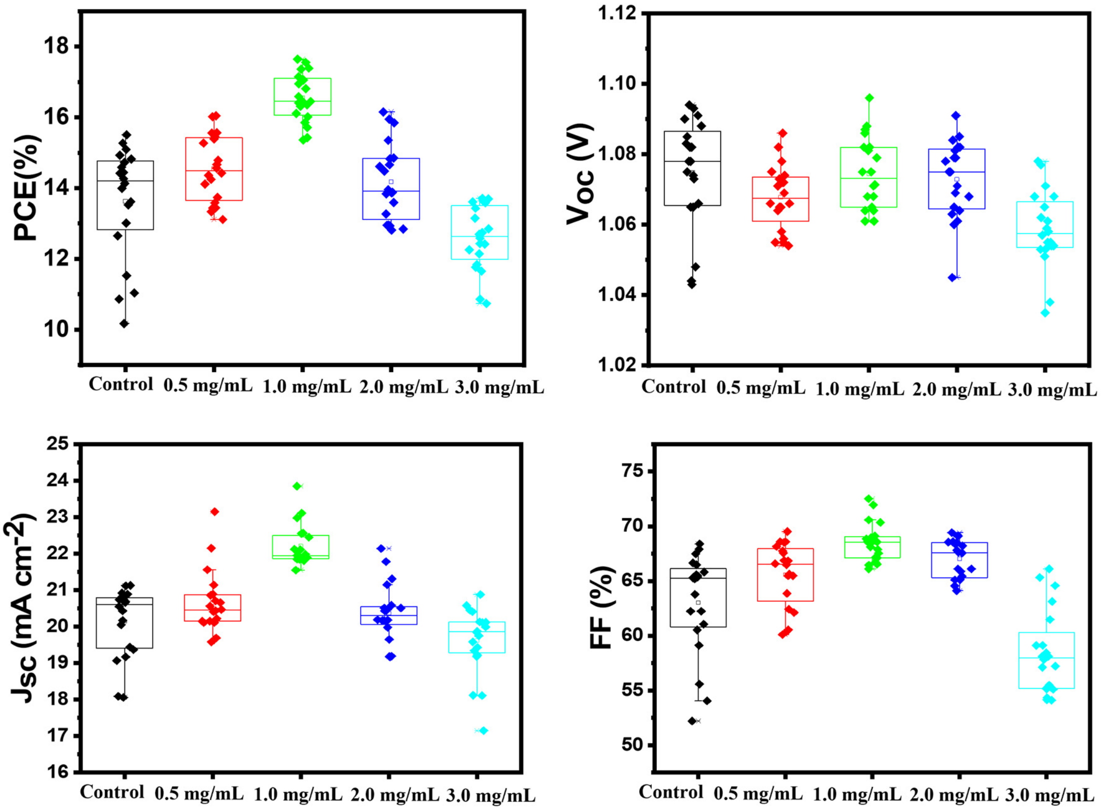

Fig. S12. Effects of varying PAA concentration on the photovoltaic parameters, including (a) PCE, (b) V_{OC}, (c) J_{SC}, and (d) FF.

Table S1. Device performance of perovskite solar cells with SnO_2 or PAA@SnO_2

**Devices**	**PCE (%)**	**J_{sc} (mA cm^{-2})**	**V_{oc} (V)**	**FF (%)**
Pristine SnO_2	15.507	21.86	1.05	63.2
0.5 mg/mL PAA	16.046	23.15	1.08	69.5
1.0 mg/mL PAA	17.145	23.85	1.08	72.5
2.0 mg/mL PAA	16.136	22.15	1.05	71.2
3.0 mg/mL PAA	13.821	19.82	1.01	62.2

Table S2. The parameters of 20 devices on pristine SnO_2 and PAA@SnO_2 ETLs.

**SnO_2**	**PCE (%)**	**V_{OC} (V)**	**J_{SC}(mA cm^{-2})**	**FF**
1	14.441	1.074	20.69	0.679
2	11.039	1.073	21.12	0.684
3	12.652	1.082	20.92	0.675
4	11.528	1.065	20.17	0.665
5	13.526	1.078	20.78	0.652
6	14.13	1.082	20.81	0.655
7	13.995	1.085	20.74	0.667
8	13.615	1.078	20.43	0.656

9	10.864	1.075	20.55	0.653
10	13.012	1.066	21.13	0.658
11	14.421	1.043	20.73	0.654
12	14.928	1.048	20.89	0.622
13	14.721	1.065	20.66	0.591
14	14.819	1.091	19.44	0.611
15	10.173	1.094	20.05	0.634
16	15.091	1.090	19.07	0.622
17	15.506	1.088	19.37	0.541
18	14.590	1.093	19.17	0.556
19	14.271	1.083	18.09	0.522
20	15.277	1.044	19.06	0.605
**Mean**	**13.629**	**1.075**	**20.14**	**0.630**
**PAA@SnO_2**	**PCE (%)**	**V_{OC} (V)**	**J_{SC}(mA cm^{-2})**	**FF**
1	16.451	1.086	23.85	0.725
2	15.851	1.082	23.11	0.720
3	17.145	1.087	22.98	0.706
4	17.056	1.079	22.45	0.704
5	15.583	1.075	22.56	0.691
6	16.456	1.081	22.11	0.685
7	15.357	1.096	22.55	0.687
8	16.112	1.075	22.12	0.689

9	13.352	1.071	21.86	0.686
10	17.641	1.082	23.55	0.686
11	16.312	1.088	21.94	0.681
12	16.415	1.064	22.85	0.665
13	17.551	1.065	21.81	0.672
14	15.423	1.068	21.86	0.671
15	16.015	1.061	22.91	0.679
16	17.361	1.068	21.85	0.689
17	17.391	1.07	21.89	0.675
18	16.811	1.065	21.94	0.667
19	15.711	1.064	21.98	0.665
20	16.951	1.061	21.95	0.661
**Mean**	**16.54**	**1.074**	**22.41**	**0.685**
